# Ceiling Effects in Satisfaction-Based Survey Items: A Psychometric Diagnostic Study of Instructional Facilitation Measures Among Indonesian Pre-Service Science Teachers

**DOI:** 10.12688/f1000research.187984.1

**Published:** 2026-09-03

**Authors:** Febrian Virijai, Eka Cahya Prima, Fitri Khoerunnisa, Rika Rafikah Agustin, Dens E.S.I. Asbanu, Resti Warliani, Hermansyah Hermansyah, Andreas Suparman

**Affiliations:** 1 Science Education, Universitas Pendidikan Indonesia, KOTA BANDUNG, JAWA BARAT, 40154, Indonesia; 2 Chemistry, Universitas Pendidikan Indonesia, KOTA BANDUNG, JAWA BARAT, 40154, Indonesia

**Keywords:** Ceiling effect, item discrimination, student satisfaction survey, psychometric diagnostics, pre-service science teachers

## Abstract

**Background:**

Self-reportsatisfaction items are widely used in large-scale educational needs-assessment surveys, but their ability to discriminate among respondents is rarely tested as a distinct research question. This study examined ceiling effects in items measuring satisfaction with instructional facilitation, spanning lecture implementation, teaching material use, and science practicum, among 348 pre-service science teachers from 29 higher education institutions in Indonesia, and their consequences for detecting a relationship with learning motivation.

**Methods:**

Analysis proceeded in four stages using established ceiling-effect criteria and item-discrimination diagnostics: distributional diagnostics against the 15% ceiling threshold, corrected item-total correlation against a validity benchmark, Pearson correlation between facilitation and motivation items with Benjamini-Hochberg correction and two-one-sided equivalence testing, and Kruskal-Wallis comparisons across five demographic variables.

**Results:**

Facilitation items were near-universally affected by severe ceiling effects, with response distributions compressed toward the scale maximum and significantly non-normal, near-bimodal kurtosis. Despite this restriction, facilitation items retained weak but consistently positive item-total correlations. Correlational analysis, supported by equivalence testing, revealed a negligible relationship between facilitation satisfaction and learning motivation at the item level. This pattern held across study program, semester, gender, age, and academic achievement groups.

**Conclusions:**

The ceiling effect reflects a systemic property of the measurement approach rather than a condition specific to any respondent subgroup. Uniformly high satisfaction scores should not be interpreted at face value in large-scale survey research. This study offers a replicable diagnostic framework, interpretable alongside Herzberg’s hygiene-motivator distinction, for identifying ceiling-affected items before their use in relational or predictive analyses.

## 1. Introduction

Self-report satisfaction surveys are among the most widely used instruments in educational quality assurance, informing decisions ranging from curriculum revision to accreditation reporting. Their use rests on an assumption rarely tested explicitly: that items retain sufficient variance to distinguish genuinely satisfied respondents from indifferent ones ( Boateng et al., 2018). When this precondition fails, a well-documented artifact emerges: the ceiling effect, whereby responses cluster at the upper end of a scale, compressing genuine variation ( McHorney & Tarlov, 1995). Originally identified in health-status measurement, ceiling effects were formalized as a quality criterion, with more than 15% of respondents selecting the maximum value indicating compromised measurement ( Terwee et al., 2007). The consequence is not cosmetic, since a compressed distribution mechanically attenuates any correlation the item might have with other variables, regardless of whether a genuine relationship exists ( Terwee et al., 2007). The broader Likert-scale literature reinforces this concern: response-option design substantially influences reliability and susceptibility to bias ( Kusmaryono et al., 2022); information loss in Likert-type data obscures underlying variation even under sound design ( Westland, 2022); and these concerns compound for reverse-scored items used in the present instrument, which are prone to misprocessing, reduced reliability, and distorted factor structure ( Weijters & Baumgartner, 2012).

Evidence that ceiling effects extend into contemporary higher education research is accumulating. A twelve-year longitudinal analysis of a national student survey documented responses persistently “moving from threes to fours,” rendering year-on-year gains largely uninterpretable ( Langan & Harris, 2019, 2023). In health-service contexts, adjusting for social desirability has been shown to reduce ceiling effects and increase variability in patient-reported measures ( Badejo et al., 2022), while comparisons of Likert and item-specific response formats find ceiling effects regardless of format ( Aletras et al., 2025), and even redesigned Guttman-type scales reduce but do not eliminate the problem ( Versluijs et al., 2020). Satisfaction surveys also carry biases unrelated to the construct measured: instructor gender confounds satisfaction ratings independent of teaching quality ( Khokhlova et al., 2023; Marques et al., 2024), and social desirability systematically biases self-reported ratings more broadly ( Zheng et al., 2023). This concern is amplified in collectivist cultural settings, where socially desirable responding is more pronounced on items touching authority figures or social harmony ( Teh et al., 2023) directly relevant to Indonesian higher education, where entering teacher education is itself often shaped by familial expectation as much as vocational interest ( Suryani, 2021).

Theory offers a further reason to expect facilitation-type items to behave differently from competency-type items. Herzberg’s two-factor theory distinguishes hygiene factors, conditions whose presence prevents dissatisfaction but does not generate motivation, from genuine motivational factors rooted in competence and growth ( Herzberg, 1968); applied to higher education, teaching methods and facilities function as hygiene factors while competence acquisition drives satisfaction and loyalty ( De-Juan-Vigaray et al., 2024). If facilitation operates as a hygiene factor here, limited association with motivation would be expected independent of measurement artifact consistent with self-determination theory’s distinction between contextual support and the proximal psychological needs driving motivation ( Ryan et al., 2022), with meta-analytic evidence that motivation is a weaker predictor of academic outcomes than commonly assumed ( Walker et al., 2024), and with review evidence that teacher satisfaction–achievement links are highly context-dependent ( Hoque et al., 2023). Robust item discrimination a corrected item-total correlation of at least 0.30 is a complementary quality criterion ( Boateng et al., 2018), which items may fail through poor construct alignment or inattention rather than ceiling effects alone ( Meade & Craig, 2012; Ward & Meade, 2023); recent validation work self-efficacy scales ( Kolil et al., 2023), Rasch-based science-teaching instruments ( Welter et al., 2024), mathematics self-direction scales ( Lin & Chen, 2023), and cross-cultural teaching-evaluation scales ( Villar-Guevara et al., 2024) confirms item-level diagnostics are now an expected standard.

A companion investigation of the same dataset (348 pre-service science teachers, 29 Indonesian institutions; cf. Hidayat et al. (2023)) found facilitation quality rated uniformly favorably, notable given that STEM education’s impact is typically heterogeneous rather than uniform ( Cao et al., 2025). Whether this reflects genuine quality or measurement artifact, that study’s descriptive design could not answer; the distinction matters because facilitation scores are routinely used in institutional research ( Okoye et al., 2022) to infer relationships with outcomes like motivation, where an undiagnosed ceiling effect would produce a null finding indistinguishable from a genuine absence of relationship. This study addresses that gap through a systematic psychometric diagnosis benchmarked against established quality criteria, investigating: first, whether facilitation items exhibit ceiling effects and compromised discrimination; second, whether this coincides with a negligible facilitation–motivation relationship; and third, whether this pattern is subgroup-specific or universal, contributing a replicable diagnostic framework for self-report satisfaction items relevant to educational survey research broadly.

## 2. Methods

### 2.1 Research design

This study employed a psychometric diagnostic design examining the measurement behavior of self-report items, rather than the substantive level of a construct, an approach following established precedent in survey methodology research that treats item-level metric properties as the primary object of analysis ( Langan & Harris, 2019, 2023). The design directly addresses three research questions, each mapped to a specific analytical stage below: RQ1: Do Facilitation items exhibit ceiling effects? RQ2: Does this affect the detectability of a Facilitation–Motivation relationship? RQ3: Is this pattern universal or subgroup-specific? This study’s evidentiary standard for “ceiling effect” rests on established normative thresholds from the psychometric literature ( Boateng et al., 2018; McHorney & Tarlov, 1995; Terwee et al., 2007) rather than an internal comparison group within this dataset, a design choice intended to avoid conflating two independently reportable findings within a single manuscript.

### 2.2 Data verification

Prior to analysis, the dataset underwent a verification procedure to confirm structural integrity. The source file contained two aggregate summary rows, comprising item-level and domain-level means, appended below the final respondent record; these rows were identified through row-by-row inspection and explicitly excluded to prevent their misclassification as respondent data. The resulting dataset of 348 respondents was confirmed to contain zero missing values in the demographic fields used for sample verification.

### 2.3 Participants, time, location, instrument

Participants, sampling procedure, data collection timeframe, and geographic coverage were identical to those reported in the companion study, which involved 348 pre-service science teachers across 29 institutions and 26 provinces during the 2025/2026 academic year. This study draws on 28 items spanning four domains: Lecture Implementation with 8 items, Teaching Material Use with 8 reverse-scored items, Science Practicum with 6 items, and Learning Motivation with 6 items, all measured on a 4-point Likert scale.

### 2.4 Data analysis

Analysis proceeded in four stages. Stage 1, addressing RQ1, evaluated each item against the ceiling-effect criterion of McHorney & Tarlov (1995), later formalized as a standard quality benchmark ( Terwee et al., 2007), whereby more than 15% of respondents selecting the maximum value indicates a ceiling effect. Skewness and excess kurtosis were tested against each estimate’s standard error, following large-sample interpretive guidance ( Kim, 2013), to formally confirm departure from normality rather than relying on descriptive magnitude alone. Stage 2, also addressing RQ1, computed corrected item-total correlations within each domain against the r ≥ 0.30 validity threshold ( Boateng et al., 2018), assessing whether ceiling-affected items retained internal coherence despite restricted variance. Stage 3, addressing RQ2, computed Pearson correlations between all 22 Facilitation and 6 Learning Motivation items, yielding 132 pairwise combinations, with p-values corrected via the Benjamini-Hochberg False Discovery Rate procedure ( Benjamini & Hochberg, 1995). A two-one-sided test of equivalence, or TOST, was conducted on the mean correlation against a ± 0.10 margin, following recommended practice for formally evidencing a negligible effect rather than relying on non-significance alone ( Lakens, 2017; Lakens et al., 2018), consistent with growing precedent for comparing effect sizes against practically meaningful bounds in educational research ( Ponce-Renova, 2022). Stage 4, addressing RQ3, tested a composite Facilitation score across five demographic variables using the non-parametric Kruskal-Wallis test ( Kruskal & Wallis, 1952), appropriate for the ordinal scale of the underlying items. Effect size was calculated via epsilon-squared, a measure increasingly reported alongside Kruskal-Wallis tests in comparative educational and health-professions survey research ( Pajari et al., 2025), with Benjamini-Hochberg FDR correction applied throughout.

### 2.5 Statistical power consideration

Given the asymmetric item counts between the Facilitation domain (22 items) and the Learning Motivation domain (6 items), post hoc power for item-level group comparisons was estimated using a normal approximation to the Mann-Whitney U distribution. With sample sizes of 22 and 6, statistical power reaches conventionally adequate levels above 0.80 only for large effect sizes (r ≥ 0.80); moderate effects would likely go undetected at these sample sizes. This limitation is explicitly acknowledged: findings involving the Learning Motivation domain as a comparison group are reported only when observed effect sizes substantially exceed this threshold and are interpreted with corresponding caution in the Discussion.

### 2.6 Validity and reliability

Response-quality indicators, comprising intra-respondent standard deviation, longstring index, and unique-value count, were re-examined using the framework established in the companion study ( Meade & Craig, 2012; Ward & Meade, 2023) to confirm that observed ceiling effects reflect item-level measurement properties rather than individual-level careless responding. No evidence of systematic careless responding was found, reinforcing that the ceiling effect originates from item design and response-context factors rather than respondent disengagement. This pattern is discussed further in light of cross-cultural evidence on socially desirable responding in collectivist survey contexts ( Teh et al., 2023). All data processing and analysis were conducted in Python 3.12 using pandas, numpy, scipy.stats, and statsmodels.

Figure 1 summarizes this four-stage analytical sequence, situating each stage’s inputs, criteria, and outputs within the overall diagnostic workflow described above.

**Figure 1.**
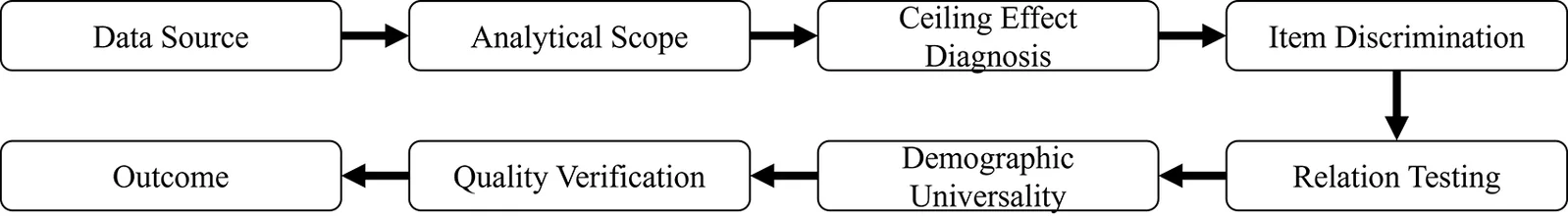
Four-stage diagnostic workflow. The analytic sequence proceeds from the shared 348-respondent dataset through definition of the analytical scope, ceiling-effect diagnosis (Stage 1), item discrimination screening (Stage 2), relation testing between Facilitation and Learning Motivation items (Stage 3), and demographic universality testing (Stage 4), with response-quality verification applied as a cross-cutting check before the outcome is interpreted.

As Figure 1 illustrates, the four stages proceed sequentially from the shared 348-respondent dataset through ceiling-effect and discrimination diagnostics to relational and demographic testing, with response-quality verification operating as a cross-cutting check applied independently of the four main stages, a design intended to rule out individual-level explanations before item-level ceiling effects are interpreted as a property of instrument design.

## 3. Results

This section presents findings across the four analytical stages outlined in Section 2.4, beginning with the distributional diagnostics that motivate the remainder of the analysis.

### 3.1 Ceiling effect and distributional diagnostics

Table 1 below summarises the distributional characteristics of all 28 items across the four domains under investigation, providing the empirical basis for the ceiling-effect classification central to this study’s first research question.

**Table 1.** Domain-level distributional diagnostics. Item counts, means, standard deviations, and ceiling-effect percentages by domain, with the number of items exceeding the 15% ceiling-effect threshold ( McHorney & Tarlov, 1995; Terwee et al., 2007) and excess kurtosis values indicating departure from normality.

Domain	Items	M	SD	Ceiling %	Kurtosis	Items exceeding ceiling threshold
Lecture Implementation	8	3.503	0.500	50.3	−1.993	8/8
Teaching Material Use	8	3.500	0.509	50.4	−1.608	8/8
Science Practicum	6	3.490	0.509	49.4	−1.769	6/6
Learning Motivation	6	2.625	0.847	15.2	−0.619	2/6

As Table 1 shows, items measuring satisfaction with Lecture Implementation, Teaching Material Use, and Science Practicum exhibited a strikingly uniform pattern: all 22 of 22 items across the three domains exceeded the 15% ceiling-effect threshold ( McHorney & Tarlov, 1995; Terwee et al., 2007), with an average of approximately half of respondents, ranging from 49.4% to 50.4%, selecting the maximum scale value. This magnitude is considerably more severe than ceiling effects documented even in health-service satisfaction contexts, where comparable instruments have reported ceiling proportions in the 30–40% range ( Aletras et al., 2025; Versluijs et al., 2020). By contrast, only 2 of the 6 Learning Motivation items exceeded this threshold, with a substantially lower mean ceiling proportion of 15.2%. This distributional compression was formally corroborated by a significance test of excess kurtosis against its theoretical standard error ( Kim, 2013). All 22 Facilitation items exhibited kurtosis significantly different from the zero-kurtosis expectation of a normal distribution, with p less than.001 for every item, whereas only 4 of the 6 Learning Motivation items reached this threshold. Skewness, by contrast, did not differentiate the two item sets, with 0 of 22 and 0 of 6 items reaching significance, respectively, indicating that the departure from normality characterizing Facilitation items is specifically one of excessive peakedness and boundary concentration consistent with ceiling-driven response compression rather than asymmetry in the conventional sense. This pattern of reporting item-level skewness and kurtosis as formal diagnostic evidence, rather than descriptive detail alone, follows precedent in recent scale-validation work in adjacent scientific-education contexts ( Kolil et al., 2023).

### 3.2 Item discrimination

Having established that Facilitation items are disproportionately affected by ceiling effects, this subsection examines whether this restriction compromised their internal coherence with the parent domain.

Despite pronounced ceiling effects, Facilitation items retained weak but uniformly positive item-total correlations: Lecture Implementation had a mean of 0.120, ranging from 0.046 to 0.199; Teaching Material Use had a mean of 0.165, ranging from 0.094 to 0.211; and Science Practicum had a mean of 0.170, ranging from 0.137 to 0.213, with none of the 22 items falling below zero. Learning Motivation items showed a lower mean item-total correlation of 0.044. This domain included one item with a negative coefficient of −0.042, despite the domain’s comparatively healthy variance and absence of a pronounced ceiling effect. Notably, no item in either domain reached the conventional validity threshold of r ≥ 0.30 ( Boateng et al., 2018). This indicates that while Facilitation items retain an internally consistent, albeit attenuated, signal, neither domain meets standard psychometric criteria for item discrimination in absolute terms.

Figure 2 below places all 28 items in a two-dimensional diagnostic space defined by the ceiling effect and item-total correlation, allowing the domain-level pattern reported above to be examined at the level of individual items.

**Figure 2.**
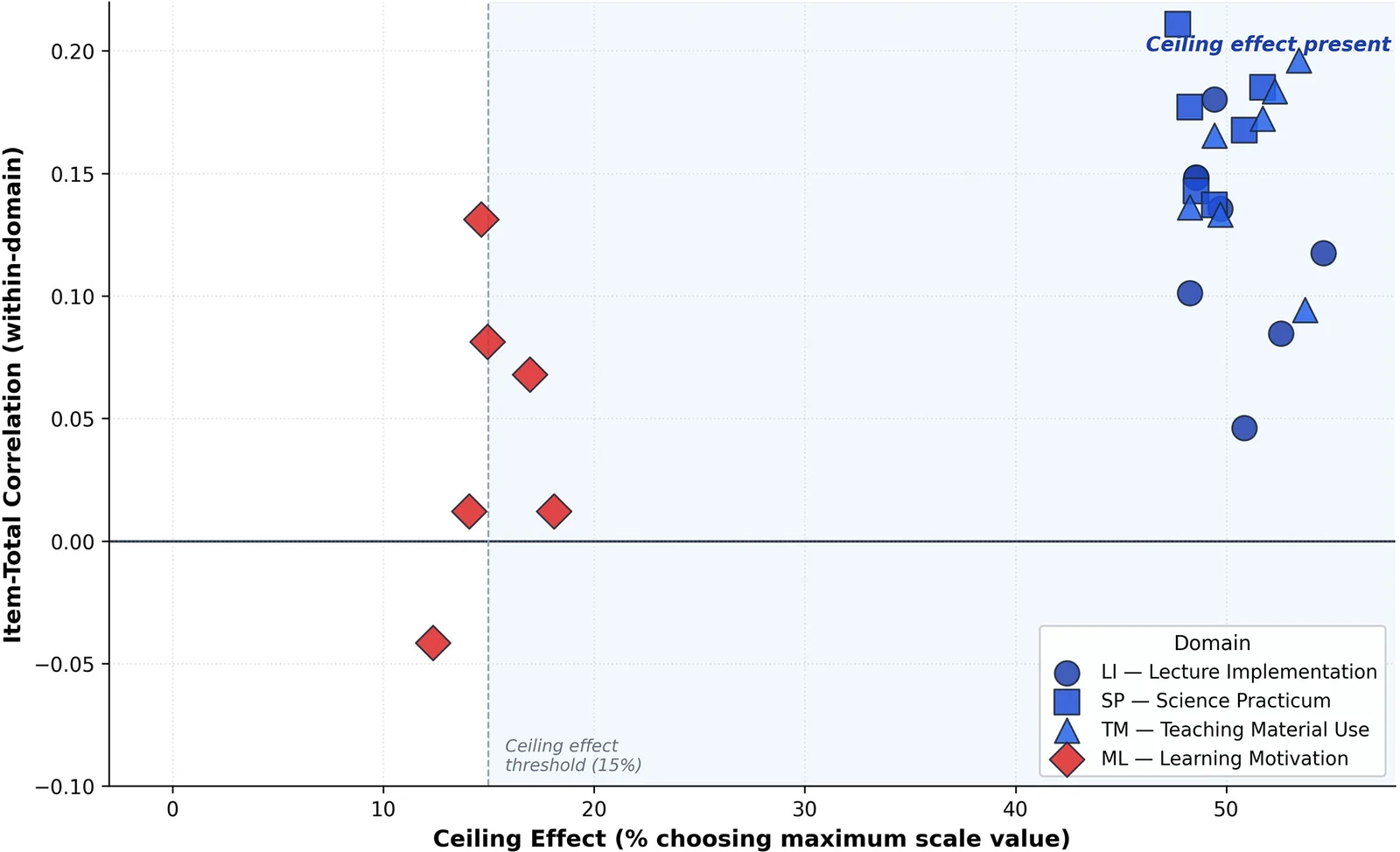
Item-level diagnostic map. Scatter plot of all 28 items positioned by ceiling effect (% of respondents choosing the maximum scale value, x-axis) against item-total correlation (y-axis). Facilitation items (Lecture Implementation, Teaching Material Use, Science Practicum) cluster above the 15% ceiling-effect threshold; Learning Motivation items cluster below it. All items fall below the horizontal validity threshold line (r = 0.30), regardless of domain.

As Figure 2 illustrates, the near-complete horizontal separation between Facilitation items, clustered above 45% ceiling, and Learning Motivation items, clustered below 20% ceiling, is visually unambiguous. In contrast, both item sets remain below the horizontal validity-threshold line, underscoring that ceiling effect and low discrimination are distinct, only partially overlapping psychometric concerns. A Mann-Whitney U comparison treating items as the unit of analysis confirmed these visual patterns held statistical support: Facilitation items differed from Learning Motivation items on ceiling percentage (U = 132.0, p < .001), item-total correlation (U = 125.0, p < .001), and kurtosis (U = 4.0, p < .001). Given the asymmetric group sizes (22 versus 6 items), these results are interpreted descriptively rather than as generalizable inferential claims, consistent with the power consideration outlined in Section 2.5.

Figure 3 below complements the item-level view in Figure 2 with a domain-level summary, permitting a more immediately interpretable visual comparison for readers less familiar with quadrant-style diagnostic plots.

**Figure 3.**
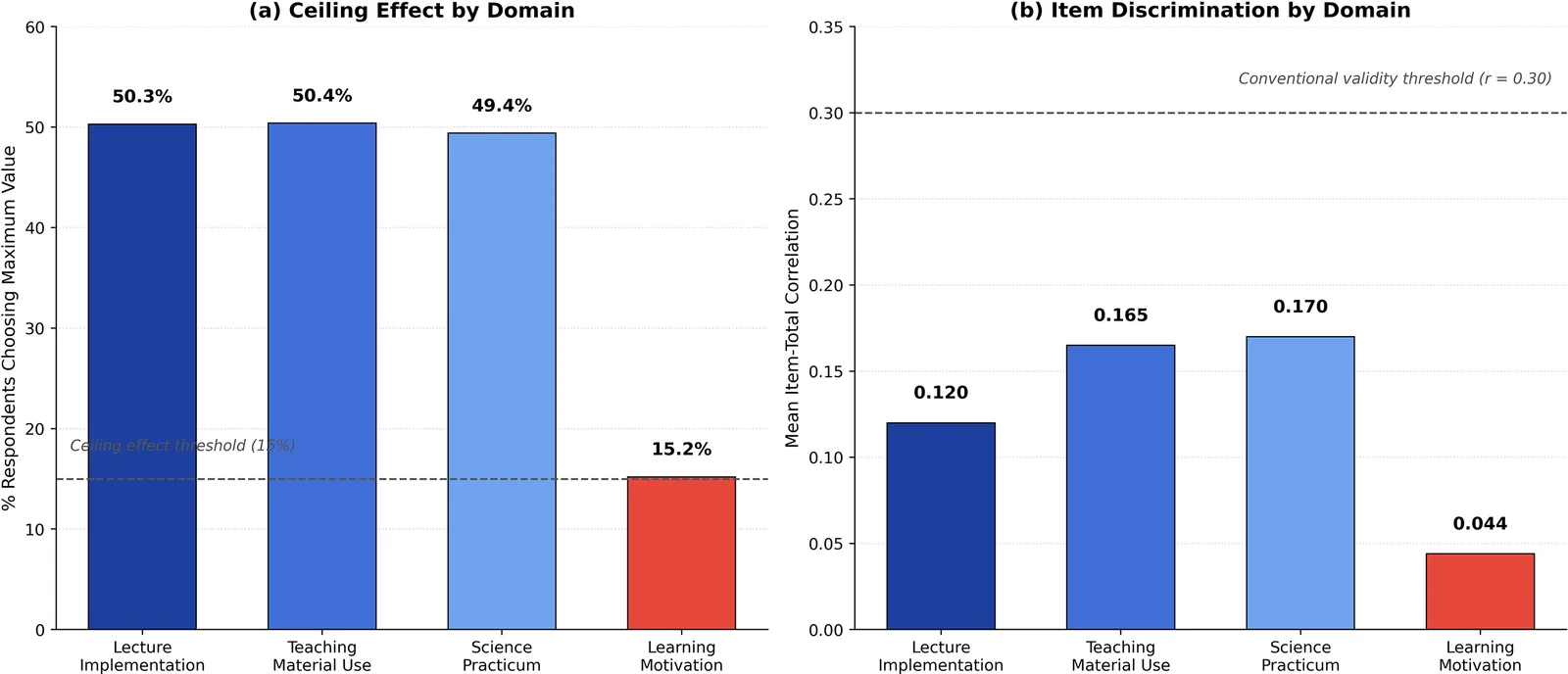
Domain-level diagnostic comparison. (a) Percentage of respondents selecting the maximum scale value by domain, relative to the 15% ceiling-effect threshold (dashed line). (b) Mean item-total correlation by domain, relative to the conventional validity threshold of r = 0.30 (dashed line). Bars show domain means; Learning Motivation (red) is shown alongside the three Facilitation domains (blue).

Figure 3 confirms, at the aggregated domain level, the same pattern established item-by-item in Figure 2: all three Facilitation domains cluster near 50% ceiling with item-total correlations in the 0.12–0.17 range, while Learning Motivation sits near the 15% ceiling threshold with a markedly lower mean item-total correlation of 0.044. The two figures are complementary rather than redundant. Figure 3 supports rapid domain-level comparison. In contrast, Figure 2 supports item-level verification of that comparison’s robustness, confirming, for instance, that the weakest-discriminating Facilitation item (Lecture Implementation, r = 0.046) still falls within the same broad cluster as the strongest (Science Practicum, r = 0.213), rather than representing a hidden outlier obscured by domain averaging.

### 3.3 Relationship between facilitation and learning motivation

This subsection addresses the study’s second research question: does the ceiling effect documented above coincide with a negligible relationship between facilitation satisfaction and learning motivation? Pearson correlations across all 132 pairings of the 22 Facilitation and 6 Learning Motivation items showed that 11 (8.3%) reached nominal significance at p < .05 before correction. However, none survived Benjamini-Hochberg FDR correction ( Benjamini & Hochberg, 1995), consistent with chance-level false positives at this sample size. Beyond testing for absence of significance, this study sought positive evidence that the relationship was negligible in magnitude rather than merely undetected: a two one-sided test (TOST) of equivalence on the mean correlation (r = −0.004, SE = 0.005) against a ± 0.10 margin, a conventional negligible-effect threshold ( Lakens, 2017; Ponce-Renova, 2022), was significant (p < .001), formally demonstrating that the true relationship lies within a negligible range rather than simply failing to reach significance due to insufficient power. This distinction matters methodologically: a non-significant result alone cannot establish absence of a relationship, whereas a significant equivalence test can ( Lakens et al., 2018).

Table 2 reports this pattern by Facilitation sub-domain, confirming the negligible relationship was not confined to any single facet of instructional facilitation.

**Table 2.** Facilitation–motivation correlations by domain. Pearson correlations between Facilitation sub-domain items and Learning Motivation items, with mean and standard deviation of correlation coefficients and the number of pairs surviving Benjamini-Hochberg FDR correction.

Facilitation Domain	Item Pairs	Mean r	SD r	Max|r|	Significant after FDR
Lecture Implementation	48	−0.010	0.061	0.130	0/48
Teaching Material Use	48	0.006	0.058	0.132	0/48
Science Practicum	36	−0.009	0.049	0.124	0/36
Overall	132	−0.004	0.057	0.132	0/132

As Table 2 shows, the near-zero mean correlation was remarkably consistent across all three sub-domains (ranging narrowly from −0.010 to 0.006), and not a single one of the 132 item pairs survived multiple-comparison correction. This uniformity across conceptually distinct facilitation domains spanning lecture delivery, instructional materials, and laboratory practicum supports the interpretation that the null relationship reflects a general property of the facilitation–motivation link in this sample, rather than an idiosyncrasy of any one measurement domain.

### 3.4 Demographic universality

This final subsection addresses the study’s third research question: is the pattern documented above concentrated within specific subgroups, or distributed uniformly across the sample?

None of the five comparisons reported in Table 3 reached significance after Benjamini-Hochberg FDR correction, and effect sizes were uniformly negligible (ε ^2^ ranging from.000 to.014), well below the “small effect” threshold conventionally applied to epsilon-squared in comparative survey ( Pajari et al., 2025). This uniform null pattern indicates that the ceiling effect documented in Sections 3.1–3.2 and the absence of a Facilitation Motivation relationship documented in Section 3.3 are not attributable to any identifiable demographic subgroup. Rather, the pattern appears to characterize the measurement approach itself, applying with comparable force across study programs, academic levels, gender, age groups, and prior academic achievement.

**Table 3.** Kruskal-Wallis demographic comparison. Test statistics, uncorrected p-values, and epsilon-squared effect sizes for the composite Facilitation score across five demographic variables, with FDR-corrected significance status.

Demographic Variable	H	p (uncorrected)	ε ^2^	Significant after FDR?
Study Programme	3.036	.386	.009	No
Semester	0.000	.997	.000	No
Gender	1.372	.242	.004	No
Age	4.843	.089	.014	No
GPA Category	3.532	.171	.010	No

## 4. Discussion

The findings carry both methodological and theoretical significance: facilitation items were nearly universally compressed toward the scale maximum, with roughly half of respondents selecting the top value across all 22 items spanning three conceptually distinct domains. This uniformity is difficult to reconcile with genuinely uniform facilitation quality across 29 institutions and 26 provinces, particularly given that STEM interventions typically produce heterogeneous, not uniform, effects ( Cao et al., 2025). The more parsimonious explanation is that the items lack sufficient discriminatory range, consistent with a twelve-year longitudinal survey documenting responses persistently “moving from threes to fours” ( Langan & Harris, 2023) and with evidence that even redesigned response formats in health-service research fail to eliminate ceiling effects ( Aletras et al., 2025; Versluijs et al., 2020), suggesting this may be a structural feature of the item format rather than a context-specific anomaly.

The negligible, equivalence-tested Facilitation–Motivation relationship invites theoretical rather than purely methodological interpretation. Herzberg’s two-factor theory distinguishes hygiene factors (conditions that prevent dissatisfaction without generating motivation) from genuine motivational factors rooted in competence and growth ( Herzberg, 1968). Applied to higher education, teaching methods and facilities function as hygiene factors, while competence acquisition drives satisfaction and loyalty ( De-Juan-Vigaray et al., 2024). If facilitation operates as a hygiene factor here, its adequacy would prevent dissatisfaction without meaningfully driving motivation, precisely the pattern observed. Self-determination theory offers a complementary account, distinguishing environmental supports from the proximal psychological needs that drive autonomous motivation ( Ryan et al., 2022). This does not exclude the ceiling-effect explanation but complements it, since hygiene-type factors are theoretically bounded in predicting motivation regardless of measurement precision. This is reinforced by meta-analytic evidence that motivation is a weaker, less consistent predictor of academic performance than commonly assumed ( Walker et al., 2024) and by review evidence that teacher satisfaction–achievement links are highly context-dependent ( Hoque et al., 2023), suggesting the assumed causal chain from facilitation through motivation to outcomes may be weaker than institutional practice presumes, independent of measurement limitations.

The cultural context warrants consideration: individuals in collectivist settings show higher socially desirable responding on items related to authority or social harmony ( Teh et al., 2023). This is relevant given that entry into Indonesian teacher education is often shaped by familial expectations ( Suryani, 2021) and that facilitation items inherently invite evaluation of lecturers and institutions. While this design cannot disentangle ceiling effects arising from item wording from those stemming from cultural response tendencies, both plausibly compound one another. This concern is not unique to collectivist settings, however: gender bias in student evaluations persists even under controlled Western conditions ( Khokhlova et al., 2023; Marques et al., 2024), and social desirability broadly distorts self-reported ratings ( Zheng et al., 2023), reinforcing diagnostic scrutiny as general practice rather than a context-specific necessity.

The ceiling effect’s demographic universality, which persists across program, semester, gender, age, and achievement, points to a property of the instrument itself rather than to a targeted subgroup explanation. This echoes arguments that aggregate satisfaction metrics risk obscuring meaningful variation without diagnostic scrutiny ( Langan & Harris, 2019) and parallels evidence that cohort-level trends can mislead absent formal measurement invariance testing ( Abulela et al., 2025). Three limitations qualify these conclusions: the cross-sectional design cannot confirm temporal stability without longitudinal replication cf. Langan & Harris (2023); the equivalence test addresses only linear associations, leaving non-linear or threshold-based relationships plausible under the hygiene-factor account untested; and the disproportionate item count (22 versus 6) constrained power for Motivation-domain comparisons, as acknowledged in Section 2.5.

Notwithstanding these limitations, this diagnostic approach offers a replicable template, consistent with growing instrument-validation practice ( Kolil et al., 2023; Lin & Chen, 2023; Villar-Guevara et al., 2024; Welter et al., 2024). Researchers and institutional administrators are encouraged to routinely apply ceiling-effect diagnostics ( Boateng et al., 2018; Kim, 2013; McHorney & Tarlov, 1995; Terwee et al., 2007) before satisfaction scores inform decision-making or predictive analyses; where a negligible relationship is suspected, equivalence testing ( Lakens, 2017; Lakens et al., 2018; Ponce-Renova, 2022) offers a more defensible tool than non-significant p-values alone helping distinguish instruments requiring revision from those genuinely capturing high-quality provision.

## 5. Conclusion

This study conducted a systematic psychometric analysis of instructional facilitation items among 348 pre-service science teachers across 29 Indonesian institutions. All 22 facilitation items exceeded established ceiling-effect thresholds, with distributions compressed toward the scale maximum and significantly non-normal, exhibiting near-bimodal kurtosis distinct from the comparatively unrestricted Learning Motivation domain, thereby answering RQ1. Despite this compression, items retained weak but consistently positive internal coherence, indicating a restricted range rather than an absent signal. Equivalence testing provided positive evidence that facilitation satisfaction bears a negligible relationship with learning motivation at the item level, addressing RQ2; this pattern held uniformly across program, semester, gender, age, and achievement, indicating a systemic rather than subgroup-specific phenomenon and thereby answering RQ3.

These findings offer three contributions. Theoretically, the negligible relationship aligns with Herzberg’s hygiene-motivator distinction and with self-determination theory’s separation of contextual support from proximal psychological needs, suggesting that facilitation may be theoretically bounded in predicting motivation, regardless of measurement precision. Methodologically, this study demonstrates that ceiling-effect diagnostics, threshold percentages, formal kurtosis testing, discrimination screening, and equivalence testing can be applied systematically to self-report items, providing a replicable framework for distinguishing genuine findings from measurement artifacts. Practically, the findings caution against interpreting uniformly high satisfaction scores as self-evident quality or using them as outcome predictors without prior diagnostic scrutiny.

Future research should prioritize longitudinal replication to test whether this ceiling effect is stable across administrations and to investigate whether revised item wording, shifting toward behaviorally specific or comparative formats, can restore discriminatory capacity without sacrificing brevity. Subsequent work might test non-linear or threshold-based facilitation–motivation models and extend cross-cultural comparative approaches ( Villar-Guevara et al., 2024) to assess whether the magnitude of the ceiling effect varies across cultural or institutional contexts. More broadly, this diagnostic sequence is offered as a low-cost addition to survey-based quality assurance practice, applicable beyond the context examined here.

## Ethical considerations

This study involved human participants, namely 348 undergraduate pre-service science teachers who completed an online self-report questionnaire. Prior to participation, all participants were informed about the purpose of the study, how their data would be collected and stored, and were provided the opportunity to give voluntary written informed consent. Participation was entirely voluntary, and participants retained the right to withdraw at any time without penalty. No sensitive personal data were collected; responses were anonymized prior to analysis, and all data are stored securely with access restricted to the research team. Formal ethical approval or a waiver from an institutional review board (IRB) was not obtained for this study, as it falls under minimal-risk, non-interventional educational research conducted within standard instructional practice, consistent with institutional research ethics practices at Universitas Pendidikan Indonesia, where ethical review is primarily required for studies involving potential risk, medical or psychological intervention, or the collection of sensitive personal data. Further information on institutional ethics practices and the role of the ethics committee at Universitas Pendidikan Indonesia can be accessed at https://etikpenelitian.upi.edu/ and https://etikpenelitian.upi.edu/berita/pentingnya-persetujuan-etik-dalam-penelitian-yang-melibatkan-manusia-di-upi. This study was designed and conducted in accordance with established principles of research ethics, including informed consent, voluntary participation, confidentiality, and protection of participant wellbeing, consistent with the Declaration of Helsinki.

## Data Availability

The dataset includes:
(1) the raw survey database in Indonesian ( *Database_ID.xlsx*), comprising 348 respondent records across 60 questionnaire items and 10 demographic variables; (2) the raw survey database in English ( *Database_EN.xlsx*), a fully translated version of the same 348 respondent records; (3) the processed dataset in Indonesian ( *Data_Processing_ID.xlsx*), containing reverse-scored item responses and computed composite domain scores for all seven constructs; and (4) the processed dataset in English ( *Data_Processing_EN.xlsx*), an equivalent English-language version of the processed data. The dataset is accessible at: https://doi.org/10.5281/zenodo.21627957 ( Virijai, 2026). The datasets supporting the findings of this study are openly available in Zenodo under a CC-BY 4.0 license. This study reports a cross-sectional survey-based needs-assessment study, adapting the STROBE (Strengthening the Reporting of Observational Studies in Epidemiology) guidelines for cross-sectional studies (von Elm, et al. 2007) to the educational survey context. Given low internal reliability observed at the domain-composite level, analysis was conducted at the item level rather than through composite scoring. Item-level significance testing employed one-sample t-tests with Cohen’s d effect sizes (Cohen, 2013), and demographic group comparisons employed the non-parametric Kruskal-Wallis test with epsilon-squared effect sizes (Tomczak & Tomczak, 2014), corrected for multiple comparisons using the Benjamini-Hochberg False Discovery Rate procedure ( Benjamini & Hochberg, 1995).
