# Supplementary material for: Ceiling Effects in Satisfaction-Based Survey Items: A Psychometric Diagnostic Study of Instructional Facilitation Measures Among Indonesian Pre-Service Science Teachers
Source: F1000Res. 2026 Sep 3;15:1477. [Version 1] doi: 10.12688/f1000research.187984.1 (PMC13736056; doi:10.12688/f1000research.187984.1)
Supplement: Peer review history

# Reviewer report: Reviewer response for version 1

**DOI:** 10.5256/f1000research.207466.r522063

**Published:** 2026-10-01

**Authors:** Renol Afrizon

**Affiliations:** 1 Universitas Negeri Padang, West Sumatra, Indonesia

**Title**

 The title is relevant and generally reflects the focus of the study. However, the term “Psychometric Diagnostic Study” may imply a broader assessment of measurement properties than is presented. The authors should consider refining the title to more precisely reflect the investigation of ceiling effects and item discrimination in satisfaction-based surveys.

**Introduction**

 The Introduction provides a relevant rationale and draws on literature concerning ceiling effects, educational satisfaction, and measurement limitations. However, the research gap and original contribution should be articulated more clearly. The authors should also distinguish the observed ceiling effects from possible explanations, such as item wording, response formats, and cultural response tendencies. The theoretical discussion would benefit from a clearer connection to the study objectives, while avoiding claims that have not been directly examined.

**Methods**

 The Methods section provides an overview of the research design, participants, instrument, and procedures. However, further details are needed to support reproducibility, particularly regarding participant recruitment, instrument development and adaptation, item wording, scoring procedures, and the rationale for the selected ceiling-effect threshold. The authors should also clarify the relationship between the items analyzed in this study and the larger dataset described in the Data Availability section. Assessment of the statistical methodology is outside the scope of this review and would require a qualified statistician.

**Results**

 The Results section is generally organized according to the research objectives and presents the main findings clearly. The reported concentration of maximum responses across the instructional facilitation items is relevant to the study's purpose. However, the authors should ensure consistency between the numerical results presented in the text, tables, and figures. Providing a complete item-level summary in the supplementary materials would also improve transparency and facilitate interpretation.

**Discussion**

 The Discussion addresses the implications of ceiling effects for educational satisfaction surveys and relates the findings to relevant theoretical perspectives. However, some interpretations appear broader than the evidence supports. The authors should distinguish empirical findings from proposed explanations and avoid attributing the observed patterns definitively to measurement design or cultural factors. The discussion of theoretical implications, demographic patterns, and practical recommendations should be more closely aligned with the study's scope and acknowledged limitations.

**Conclusions**

 The Conclusions summarize the main findings and highlight the importance of examining ceiling effects in educational surveys. However, the claims regarding the systemic nature of these effects and their implications for measurement should be expressed more cautiously. The authors should emphasize the findings directly supported by the study, acknowledge the limitations of the research context, and identify further validation across instruments and educational settings as a direction for future research.

Is the work clearly and accurately presented and does it cite the current literature?

Partly

If applicable, is the statistical analysis and its interpretation appropriate?

I cannot comment. A qualified statistician is required.

Are all the source data underlying the results available to ensure full reproducibility?

Yes

Is the study design appropriate and is the work technically sound?

Partly

Are the conclusions drawn adequately supported by the results?

Partly

Are sufficient details of methods and analysis provided to allow replication by others?

Partly

Reviewer Expertise:

Physics Education, STEM Education

I confirm that I have read this submission and believe that I have an appropriate level of expertise to confirm that it is of an acceptable scientific standard, however I have significant reservations, as outlined above.

# Response

**Published:** 2026-10-25

**Authors:** Febrian Virijai

**Affiliations:** Science Education, Universitas Pendidikan Indonesia, Bandung, West Java, Indonesia

Dear Dr. Renol Afrizon,

 Thank you for your thoughtful evaluation of our article. Your comments, together with those of Reviewer 1, guided a substantial revision. All changes are incorporated in Version 2, as summarised below.

 Title

 1.The title now reads “Ceiling Effects and Item Discrimination in Satisfaction-Based Instructional Facilitation Measures Among Indonesian Pre-Service Science Teachers,” which names the two properties actually examined. “Psychometric diagnostic study” has been replaced with “measurement diagnostic analysis” throughout.

 Introduction

 2. The final paragraph now states the research gap more clearly: whether uniformly favourable facilitation ratings reflect genuine consistency or limited item discrimination has received little attention, although such scores are often used in institutional research (Okoye et al., 2022). The contribution is defined as a focused measurement-diagnostic perspective applied before satisfaction scores are used in relational or predictive analyses.

 3. The observed ceiling pattern is now separated from its possible explanations. Item wording, response format, and cultural response tendencies are presented as candidate explanations that should not be treated as established causes unless measured directly.

 4. Herzberg’s two-factor theory and self-determination theory are now explicitly connected to the study’s second research question: they offer a possible substantive reading of weak Facilitation-Motivation associations. We state that these perspectives do not establish the mechanisms in the present study.

 Methods

 5. Section 2.3 describes participant recruitment through the researchers’ lecturer network, online dissemination via Google Forms, voluntary participation with informed consent, and eligibility criteria.

 6. Section 2.4 describes the conceptual basis and adaptation of each domain (SEEQ, LORI, SLEI, and SMQ-II), the item direction, and the reverse-scoring procedure (5 − raw response) for the eight Teaching Material Use items. Full item wording and scoring are provided in Table S1 of the Extended data.

 7. Section 2.5 explains the rationale and limits of the 15% ceiling criterion, which was originally proposed for scale scores rather than single items and is therefore used as an operational benchmark.

 8. We clarify the relationship between the analysed items and the dataset described in the Data Availability section. The 28 analysed items are a subset of a broader 60-item questionnaire covering seven constructs; only the four domains aligned with the research questions were analysed, and the archived dataset contains these 28 items together with the demographic variables.

 9. Following Reviewer 1’s statistical comments, the item-based power analysis, the item-level Mann-Whitney U tests, and the equivalence test were removed because items are not independent observations.

 Results

 10. All values in the text, tables, and figures were cross-checked. We corrected the description of kurtosis, added the corresponding procedure to Section 2.5, and corrected the ranges of corrected item-total correlations.

 11. A complete item-level summary is now provided as supplementary material in the Extended data (F1000Research’s repository-based format), in Table S3: response frequencies, means, standard deviations, skewness, kurtosis, ceiling percentages, and corrected item-total correlations for all 28 items. The distribution of respondents across institutions is provided in Table S2.

 Discussion

 12. Empirical findings are now separated from proposed explanations. We no longer attribute the ceiling pattern definitively to measurement design or cultural factors. The Discussion states that the observed pattern may reflect genuinely favourable experiences, item or format characteristics, contextual influences, or a combination of these.

 13. Theoretical perspectives are now presented as interpretive lenses rather than demonstrated mechanisms. Demographic findings are no longer described as universal, and clustering within institutions is acknowledged. Practical recommendations are limited to routine diagnostic checks of response distributions and item discrimination, and a limitations paragraph has been added.

 Conclusions

 14. The claim about the “systemic nature” of the ceiling effect has been removed. The Conclusion now emphasises the directly supported findings, acknowledges the single cross-sectional, self-report, non-probability sample, and calls for validation across different instruments, response formats, and educational settings.

 The underlying and extended data are now available at https://doi.org/10.5281/zenodo.23644011.

 We sincerely appreciate your constructive feedback and hope the revised version addresses your concerns.

 Sincerely,

 Febrian Virijai, on behalf of all authors

# Reviewer report: Reviewer response for version 1

**DOI:** 10.5256/f1000research.207466.r522060

**Published:** 2026-09-24

**Authors:** Fuja Novitra

**Affiliations:** 1 Universitas Negeri Padang, West Sumatra, Indonesia

Title

 Consider narrowing the term “psychometric diagnostic study.” The analyses primarily focus on response-distribution diagnostics, item-total correlations, and associations among variables rather than a comprehensive psychometric evaluation of the instrument. Terms such as “psychometric analysis” or “measurement diagnostic analysis” may be more proportionate to the scope of the study.

 Introduction

 The claim that a ceiling effect “mechanically attenuates any correlation” is too absolute. In principle, restriction of range can reduce observed correlations, but the use of “any correlation” is overly broad. The magnitude of attenuation depends on factors such as the distributions of the variables, the form of the underlying relationship, measurement reliability, and how the variables are constructed.

 Methods

- Please provide a clearer definition of “psychometric diagnostic design” and identify methodological sources that support the use of this terminology.
- RQ2 should be reconsidered or framed more cautiously. The data demonstrate the coexistence of substantial ceiling concentration and a negligible observed correlation, but they do not directly establish that the ceiling effect caused or attenuated the observed correlation.
- RQ3 should also avoid the term “universal.” The study examines only selected respondent characteristics and does not address all potential sources of heterogeneity, particularly variation at the institutional level.
- Please describe the sampling procedure explicitly, including how the participating institutions and respondents were recruited.
- Please report the distribution of respondents across the participating institutions so that readers can assess potential institutional imbalance.
- Because respondents are nested within 29 institutions, please acknowledge the possibility of clustering/nested data and clarify whether institutional heterogeneity could affect the interpretation of the findings.
- Please provide a more specific justification for applying the 15% ceiling threshold to item-level Likert responses, including clarification of whether the cited criterion was originally intended for interpretation at the item level.
- Please clarify that r ≥ .30 is a commonly used rule of thumb rather than a universal validity criterion. Corrected item-total correlation should also not be presented as evidence of construct validity by itself.
- Please provide more detail on how the 22 Facilitation items were developed and the conceptual basis for each domain, as the interpretation of the ceiling effect depends on the construct that the items are intended to measure.
- The power analysis requires substantial reconsideration. Using the 22 Facilitation items and 6 Motivation items as sample sizes for the power analysis should be carefully reconsidered because these items are not independent observations.
- If the inferential unit is the respondent, N = 348 should constitute the relevant sample size rather than 22 and 6. If the item-level analysis is retained, the dependence among items needs to be explicitly considered. Consider removing the post-hoc power analysis if a defensible methodological justification cannot be provided.
- Please clarify the rationale for conducting inferential Mann–Whitney U tests comparing the 22 Facilitation items with the 6 Motivation items. These items are not independent observations because they are all derived from responses provided by the same 348 participants.
- If this comparison is retained, please provide a clear methodological justification for treating items as the units of analysis and clarify the inferential status of these tests. If an adequate justification cannot be provided, consider presenting the comparison descriptively rather than using inferential p-values.

Results

- If the term “severely ceiling-affected” is retained, please provide a clear basis or criterion for classifying an item as severely ceiling-affected.
- Please avoid stating that the ceiling effect caused the negligible association. The findings demonstrate that substantial ceiling concentration and very small observed Facilitation–Motivation associations occur together, but they do not directly establish a causal relationship or quantify the extent to which the ceiling effect attenuated the observed association.

Discussion

 The Discussion would benefit from a clearer distinction between empirical findings and theoretical interpretation. Several interpretations appear stronger than warranted by the study design. In particular, the proposed explanations based on insufficient item discrimination, Herzberg’s hygiene-factor framework, Self-Determination Theory, and culturally shaped response tendencies are plausible, but these mechanisms were not directly measured or tested in the present study. Please frame them as possible explanations or hypotheses rather than mechanisms established by the data.

Is the work clearly and accurately presented and does it cite the current literature?

Yes

If applicable, is the statistical analysis and its interpretation appropriate?

I cannot comment. A qualified statistician is required.

Are all the source data underlying the results available to ensure full reproducibility?

Partly

Is the study design appropriate and is the work technically sound?

Partly

Are the conclusions drawn adequately supported by the results?

Partly

Are sufficient details of methods and analysis provided to allow replication by others?

Partly

Reviewer Expertise:

Physics Education; Teaching and Learning; Educational Technology

I confirm that I have read this submission and believe that I have an appropriate level of expertise to confirm that it is of an acceptable scientific standard, however I have significant reservations, as outlined above.

# Response

**Published:** 2026-10-25

**Authors:** Febrian Virijai

**Affiliations:** Science Education, Universitas Pendidikan Indonesia, Bandung, West Java, Indonesia

Dear Dr. Fuja Novitra,

 Thank you for your careful and constructive review. Your comments helped us align our claims with what the study design can support. All changes are incorporated in Version 2, as summarised below.

 Title

 1. The title now reads “Ceiling Effects and Item Discrimination in Satisfaction-Based Instructional Facilitation Measures Among Indonesian Pre-Service Science Teachers.” “Psychometric diagnostic” has been replaced with “measurement diagnostic analysis” throughout.Introduction

 2. The claim that ceiling effects “mechanically attenuate any correlation” has been revised. The text now states that restricted variability *can* reduce observed associations, depending on the distributions of the variables, measurement reliability, construct characteristics, and the form of the relationship (Westland, 2022).

 Methods

 3. Section 2.1 now defines measurement diagnostic analysis and states that factor structure, measurement invariance, and criterion validity were not examined.

 4. RQ2 now asks what pattern of association is observed, without implying causation or attenuation.

 5. “Universal” has been removed from RQ3, which now asks whether facilitation scores vary across the demographic subgroups examined.

 6. Section 2.3 describes recruitment through the researchers’ lecturer network, online dissemination via Google Forms, voluntary participation with informed consent, and eligibility criteria.

 7. The distribution of respondents across the 29 institutions and 26 provinces is reported in Table S2 of the Extended data.

 8. The nesting of respondents within institutions is acknowledged in Sections 2.3, 2.5, and 3.4 and in the limitations. Because institutional effects were not modelled, results are not interpreted as uniform across institutions.

 9. We clarify that the 15% criterion was originally proposed for scale scores, not single items. It is therefore used as an operational benchmark and interpreted together with the observed magnitude (approximately 50%).

 10. r = .30 is now described as a rule-of-thumb discrimination benchmark, and corrected item-total correlations are explicitly not treated as evidence of construct validity.

 11. Section 2.4 describes the conceptual basis of each domain (SEEQ, LORI, SLEI, and SMQ-II) and the adaptation of the items. Full item wording and scoring are provided in Table S1.

 12. The post hoc power analysis has been removed. All analyses are based on the 348 respondents.

 13. The item-level Mann-Whitney U tests have been removed, and the item comparisons are now reported descriptively because items are not independent observations. For the same reason, we also removed the equivalence test on the mean item-pair correlation.

 Results

 14. “Severe” has been removed. Ceiling concentration is described factually (approximately half of respondents selected the maximum category).

 15. All causal wording has been removed. Section 3.3 states that the findings do not establish that ceiling concentration caused or attenuated the associations. We also corrected the kurtosis description and the reported ranges of corrected item-total correlations.

 Discussion

 16. Empirical findings are now separated from interpretation. Herzberg’s theory, self-determination theory, item discrimination, and cultural response tendencies are presented as possible explanations that were not tested. A limitations paragraph has been added.

 The underlying and extended data are now available at https://doi.org/10.5281/zenodo.23644011.

 We are grateful for your valuable comments and hope the revised version addresses your concerns.

 Sincerely,

 Febrian Virijai, on behalf of all authors
